# Towards a Policy Development Methodology for Human-Centred IoT Collectives

**DOI:** 10.3390/s22197401

**Published:** 2022-09-29

**Authors:** Amna Batool, Seng W. Loke, Niroshinie Fernando, Jonathan Kua

**Affiliations:** School of Information Technology, Deakin University, Geelong, VIC 3220, Australia

**Keywords:** methodology, socio-ethical policies, IoT collectives, human-centred IoT

## Abstract

Embedding ethical concepts into smart Internet-connected devices and making them behave in a more human-centred manner, i.e., ethically and in a socially acceptable manner, has received significant attention in the software industry. To make smart devices behave in more human-centered manners, it is important to develop a methodology for defining smart devices’ key roles and mapping them with socio-ethical and administrative policies. This paper proposes a policy development methodology for making smart devices more human-centred by following its four phases i.e., concept development, defining and mapping policies, implementing the processing of policies, and deploying the devices. The suggested methodology may be used in a variety of situations where smart devices interact with people. For illustration, the proposed methodology has been applied to three different settings, including a supermarket, a children’s hospital, and early learning centers, where each phase defined in the methodology has been followed. The application of the methodology to smart internet-connected devices, including robots, smart cameras, and smart speakers, has shown significant results. It has been observed that the devices behave in more human-centric ways while performing their core functions, adhering to socio-ethical policies.

## 1. Introduction

The Internet of Things (IoT) can be defined as a network of Internet-connected objects, or ‘smart devices’ with computing, sensing and/or actuating capabilities. The usage of these smart devices within diverse contexts such as smart homes, supermarkets, farms, hospitals and aged-care settings are increasingly becoming popular [1]. In such environments, multiple users can use smart devices to interact with collections of other smart devices (e.g., family members living in a smart home). In this work, the term IoT collectives is used to refer to such systems [2]. IoT collectives can support ecosystems consisting of different users and devices such as smartphones, robots, drones and wearable devices to interact and work together. However, managing the interaction behaviours of a collection of heterogeneous devices, and ensuring responsible conduct, in a human-centric way remains a challenge. Consider a store with several smart trolleys and human customers, where a smart trolley approaches people and begins following them to carry their items without their consent. When the smart device engaged with the user in this scenario, it executed its core functions without the user’s permission. Is it appropriate for a smart device to behave in this manner?

There is a growing body of literature investigating how people react towards smart devices [3,4,5,6]. The noteworthy research is on how to make smart devices behave more human-centrically using ethical frameworks. In one study, Dylan and Aimee [7] proposed an ethical framework to make the design, development, deployment, and evaluation of drones in public healthcare more efficient. The framework is founded on ethical concepts such as beneficence, non-maleficence, autonomy, and fairness, as well as comprehensibility. The ethical framework of the study connects abstract ethical concepts to human values, and then connects them to design needs through a translation process using contextual norms. Unfortunately, the study only targeted drones in healthcare centers, whereas, this paper has proposed a generalised methodology which is applicable to collections of heterogeneous smart devices in a variety of domains, not only healthcare centers. Another approach is used in [8] which is the value-sensitive design method to solve an engineering design challenge involving the creation of a command and control supervisory display for the Tactical Tomahawk missile. VSD is an engineering education tool that bridges the gap between design and ethics for many engineering disciplines. Although VSD is highly recommended in engineering, it does not commit to a certain ethical framework/theory [9].

In another study [10], Van Wynsberghe suggested Care Centered Value Sensitive Design approach for infusing ethics into the robot design process (CCVSD). The study provided a preliminary analysis of how the CCVSD technique may be used to evaluate personal and professional service robots. CCVSD’s normative underpinnings stem from its dependence on the care ethics tradition, namely the use of care practices for (1) organising the analysis and (2) defining the ethically significant values. The CCVSD technique is primarily intended for use in the healthcare sector, but the study has recommended that it be used for other service robots and has found it valuable to assess if the CCVSD approach’s ethics care practices can be applied to service robots.

The main ethical principles of medicine were stated by Feil-Seifer and Matarić [11] as a platform for debating ethical difficulties raised by the Socially Assistive Robots technologies now being developed for elderly and children’s care. The study pointed out that because this ethical framework was developed using US ethical rules, it is feasible that other cultures would face different or extra ethical issues. More research is needed, particularly to see if robots developed and proven in one medical care system might act ethically in another. Furthermore, because users’ attitudes to robots range from one group to the next, ethical concepts that work for one user demographic may not work for another.

This work focuses on controlling the interaction behaviours of such smart devices that are capable of performing autonomous functions. To do so, the approach proposed in this paper is to identify the primary functions performed by smart devices, then map those functions to applicable socio-ethical and administrative policy rules, and finally embed the policy rules onto these devices, to operationalise the behaviour of smart devices. To this end, for developing an application that involves multiple IoT devices, i.e., involving IoT collectives, this paper’s main contribution is to propose a methodology that consists of four phases to guide developers, throughout the design and development process, in thinking about the desired socio-ethical behaviours of IoT devices and the corresponding organising-ethical policy rules that represent those desired socio-ethical behaviours. This paper is an extension of the conference paper [12]. This paper proposed methodology includes modified/new phases (i.e., concept development, and the addition of a demographics step) that are applied to new scenarios (from scratch) including children’s hospital, early learning center, and supermarket; whereas the conference paper only applies the methodology to the supermarket scenario.

Authorisation, obligation, and prohibition rules are the three types of rules that govern the behaviour of smart devices in socio-ethical and administrative policies [2]. These rules are intended to apply to a variety of smart devices in IoT collectives. The socio-ethical policy rules are intended to be applicable across many domains, such as supermarkets, education centres, and elderly care facilities, while the administrative policy rules are particular to the environment. Each rule under socio-ethical and administrative policy is spelled-out explicitly in the XML language to make the rules operationalisable by smart devices. There are three policy language documents that have been designed for each scenario to be processed by IoT devices to learn what policies to follow and how to interpret each policy rule while doing their respective core operations.

The approach proposed in this paper encourages a separation of concerns when thinking about IoT collectives—first identify core functions of IoT devices as one concern, and then, the socio-ethical aspects as another concern, i.e., identify the associated authorisations/prohibitions and obligations that would be associated with these core functions. The proposed methodology in this work has been applied to three different scenarios, where the prototypical futuristic environments of a supermarket, a children’s hospital, and early learning center are outlined, with multiple smart devices installed. This is explained in detail later in this paper.

How to embed AI ethical concepts into device software is now a hot topic [13]. The suggested approach is a means to incorporate ethics into smart devices through defined socio-ethical rules and make them act ethically, which contributes an approach (albeit a pragmatic and computational one) towards addressing this issue. Software developers will benefit from the methodology because they will be able to create human-centric smart devices that conduct operations ethically. Normally, software developers use software engineering approaches to create software for a specific device, but the methodology proposed in this paper will let them create ethical devices that act correctly in different situations.

The rest of this paper is organised as follows. Section 2 presents the background and related work. Section 3 presents the reference architecture. Section 4 presents an overview of the methodology for human-centred IoT Collectives. Section 5 presents the application of the proposed methodology to the three different scenarios. Section 6 outlines the discussion and Section 7 concludes with an outline of future work.

## 2. Background and Related Work

Smart devices are utilised to generate human–device interaction, but little effort has been made to make these devices behave socio-ethically. Different approaches for making AI responsible have been presented in the literature, but they are primarily centred on single smart devices, not IoT collectives. Zhu et al. [13] looked at three challenges linked to human and AI interaction, which are hard-to-implement ethical AI principles, the generic idea of trust versus trustworthiness, and product/process support for trust/trustworthiness. In the context of AI ethical principles and responsible AI, the study highlighted the missing components in operationalising ethical AI concepts and potential solutions by utilising the example of agricultural yield prediction. However, aside from trust and trustworthiness, the study did not address other aspects of ethical standards. The methodological approach proposed in this paper involves a phase of mapping socio-ethical policies, which include authorisation, obligation, and prohibition rules, to the fundamental tasks of devices in order to make them behave responsibly (or socio-ethically). Furthermore, the proposed methodology enables the operationalisation of ethical smart device behaviour using policies. In addition, the proposed approach does not address ethical AI in general, but specifically, smart devices (which might have embedded AI functionality). It is essential that several strategies or approaches be considered in order to make smart devices responsible or ethical, but it is also necessary to examine smart device behaviour in many fields. Numerous studies in the literature have concentrated on the acceptance of smart devices for usage in various domains, such as smart homes, aged care centres, supermarkets, or making them interact with humans in these settings [14,15,16,17], but none have focused on devices’ socio-ethical behaviour.

### 2.1. IoT/Robots in Supermarkets

Iwamura et al. [18] addressed the use of assistance robots by elderly people in supermarkets. The research team created two types of robots: a cart robot that can carry items and a conversational robot that can speak with elderly people as they shop. Twenty-four older citizens were invited to engage with robots at a supermarket as part of an experiment. Both robotic designs were approved by the seniors, but they preferred the talking one. The study focused on the design of robots rather than on their ethical behaviour with the elderly. This study focuses on controlling the behaviour of smart devices through policies imposed on them using the proposed methodological approach. Murshed et al. [19] introduced the EdgeLite concept, which enables smart devices to identify and react to hazards on supermarket floors in real time. The researchers created real-world collection of pictures depicting dangers on store floors. This collection includes pictures of supermarkets that have been classified as having a dangerous floor and not. This work focuses on controlling devices in environments, e.g., in supermarkets using policies.

Wang and Yang [20] presented a 3S-cart sensor-based supermarket cart for smart shopping. The 3S-cart can track the actions of its users and respond accordingly. Customers can utilise the LCD screen on the sensor-based cart to request product details, or the 3S-cart can monitor user behaviour and automatically show item data on the screen. The sensor-based cart is an IoT-based project, and while it is fascinating to observe how it detects users’ behaviour, the proposed approach in this paper/research is focused on device behaviour towards people.

Aaltonen et al. [3] tested how people react to seeing a Pepper (a social humanoid) robot at a store. Pepper was a success with both kids and adults as it sought to make and maintain eye contact with them, answered simple questions such as "How old are you?" posed for a photo, and shook hands. The authors were able to investigate the robot’s behaviour in shopping malls, including how it initiated discussion, spoke in the customer’s native language, and provided various services.

Cheng et al. [21] proposed the design and implementation of a service robot with three types of services to provide to customers in a supermarket. The robot’s first service is to execute clients’ orders by locating, picking up, and placing the item in the basket. The second service is following the client and providing assistance as needed, while the third service entails moving about to serve customers as needed. The results revealed that the service robot delivers practicable and intelligent interaction, as well as a convenient and comfortable shopping experience for the consumer.

Lewandowski et al. [22] presented a system for social navigation and human-robot interaction for a mobile robot using an out-of-stock detection technique. The technology categorises the scenarios that arise during scanning so that a mobile robot may respond to customers respectfully. The robot maintains as much distance from people as possible by employing anticipatory waiting and an asymmetrical social space. Information is communicated to a wide audience and consider accessibility for those with impairments using speech and display outputs, a video projector, and an LED Omni light.

Different robots have been utilised to develop human–device interaction for various age groups, but no research has concentrated on incorporating ethics into such robots to make them behave in more human-centred approach. The literature on IoT in supermarkets has looked at a variety of methods to make shopping easier for customers, but incorporating ethics into IoT devices can make the interaction more comfortable and convenient.

### 2.2. IoT/Robots in Healthcare

Bloss [23] explored how mobile robots might help hospitals with a range of logistical issues. He spoke with the Aethon hospital’s mobile robot logistics system developers to learn more about how mobile robots are helping the hospital’s logistics. The findings showed that mobile robots may significantly enhance hospital logistic services, such as carrying food, lab samples, and medicines, while also providing some enjoyment.

Dawe et al. [24] conducted a survey on social robots, which are utilised in healthcare to assist children. The review comprised seventy-three publications. The review’s final finding was that the NAO was the most regularly employed robot in children’s hospitals. Overall, social robots show great promise and have the ability to aid children in hospital settings, but more study is needed, including experimental methods and bigger sample sizes. The study determined the acceptability of social robots by children in healthcare, while this paper is determining the ethical policies that are applicable to the smart devices, including robots that interact in more human-centric manners with the users of any age group.

Kimura et al. [25] used a robotic pet to conduct a study at a children’s hospital to see how robot-assisted activity (RAA) affected children’s moods (AIBO). The experiment demonstrated that the RAA improves the mood and mental health of children significantly. Furthermore, there was evidence of enhanced communication among kid inpatients or nurses. It was determined that by efficiently introducing human-to-human (companion) contact, robotic–human (subject; child inpatient in this study) interaction was emphasised.

The literature on IoT/robots in children’s hospitals focused on making robot–child interaction convenient. However, it is vital to give some effort to integrating ethics into the robots used to engage with children. There are a variety of robots that children like engaging with, such as AIBO and NAO, which indicate that children prefer interacting with robots over people, according to the literature.

### 2.3. IoT/Robots in Early Learning Centre/Education

The robot had been an excellent tool for capturing and maintaining children’s attention. Tanaka et al. [26] presented preliminary findings from a study aimed at evaluating a social robot algorithm in real-world, uncontrolled situations. An experiment was performed in the classroom as part of the investigation. Every morning, there was time set out in the school for physical activity, such as dancing. It is estimated that it is not always simple to retain children’s attention on a certain job, but it has been seen that when the robot (QRIO) was present, children were more involved in the dance activity than when it was gone.

Tolksdorf et al. [27] observed ethical concerns about the use of robots in kindergarten settings. When maintaining robots in kindergarten education facilities, the authors listed three characteristics that must be considered: privacy, security, and liability. More research on the ethics of child-–robot interaction and development processes is needed, according to the study, when creating or assessing such technology.

Fridin [28] presented how an ethical approach might be used to a kindergarten environment’s first experience with a robot for pre-school children. The study created SAR technology, named Kindergarten Social Assistive Robot (KindSAR), for kindergarten child care. The study envisions a future in which KindSAR can meet the requirements of children from a variety of backgrounds, including those who are typically developing, those who have cognitive or motor disabilities, and others. The proposed KindSAR technology was shown to have various advantages over existing computer-based instructional aids such as computer games, virtual reality, and so forth.

According to the literature, the utilisation of robots or IoT devices in early learning centres has proven to be very beneficial to the children. The research has also focused on the adoption of various ethical techniques, although without giving an adequate solution.

### 2.4. Summary

In the literature, various approaches to making smart devices responsible have been explored, and only a few have discussed the integration of ethics in IoT, and do so without providing an effective computational solution/approach. The methodology proposed in this paper can be used to operationalise ethical behaviour in the Internet of Things by enacting policies that govern smart device interaction. Using smart devices in various areas is useful, but maintaining control over device behaviour is equally important. The proposed approach also enables devices to interact with people in a socially appropriate manner. The stages of the methodology are detailed later in this work.

## 3. Reference Architecture

**Figure 1:**
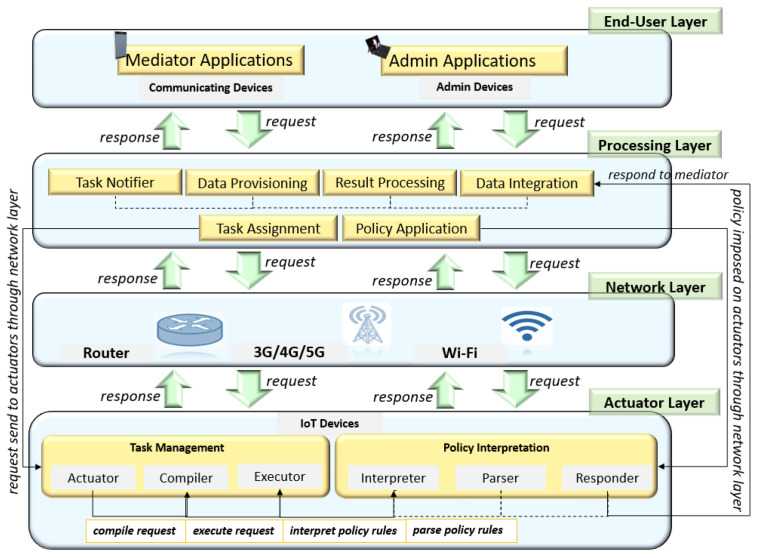
Reference architecture represents devices: communicating devices, admin devices, and IoT devices.

The proposed methodology relates to IoT devices explicitly represented in the designed reference architecture. Figure 1 presents the reference architecture consisting of four layers: end-user layer, processing layer, network layer, and actuator layer. The mediator and administrator applications that operate on communicating and admin devices, respectively, make up the end-user layer. The communicating devices are those that allow users to engage with actuators (which are IoT devices), while the admin devices are those that allow facility managers to enforce administrative policy rules on IoT devices. Some of the examples of communicating and admin devices are smartphones, laptops, desktops, tablets, and so on. The communicating devices and admin devices connect with actuators through the processing and networking layers. In the processing layer, the tasks and policies to be imposed on IoT devices generated by communicating and admin devices are handled. The tasks and policies are transferred to the Actuator layer through Wi-Fi, 3G/4G/5G, or routers, which are network layer components, once they have been processed. The IoT devices/Actuators compile and perform the job once it reaches the Actuator layer. They use the interpreter component to see if the admin has imposed policy rules on an intended task, and interpret the policy rules accordingly. When the actuators have completed their tasks, they communicate with the communication and admin devices via the network layer. The data that communicating and admin devices receive from actuator devices or vice versa, are combined, processed, and then sent to them through a task notifier as shown in the processing layer. In this manner, mediators and administrators send requests to actuators, while actuators provide replies back to mediators and administrators through the network and processing layers.

## 4. Development Methodology for Human-Centred IoT Collectives

**Figure 2:**
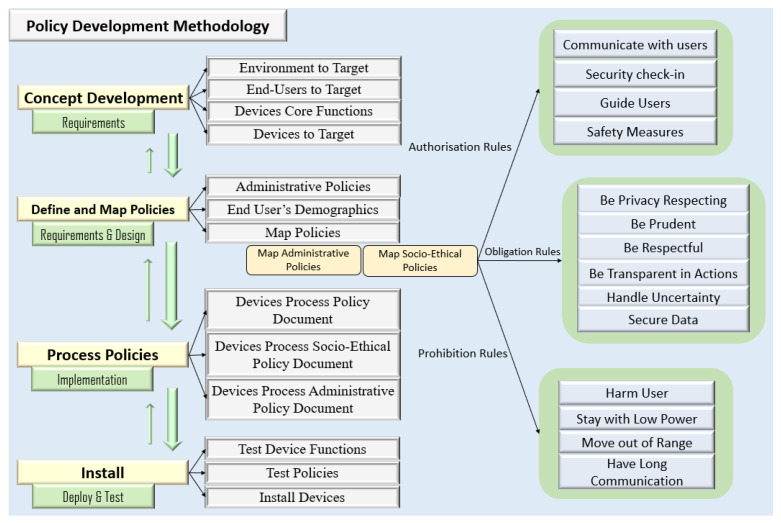
Policy development methodology for IoT collectives.

The methodology described in this paper aims to help developers make smart devices more human-centric by managing their interaction behaviour through the application of policies to them. Each stage of the recommended methodology is linked to the software engineering methodology approach. The first stage of the methodology, ‘concept development’, is similarly related to the first phase of the software development lifecycle (SDLC), requirements gathering and analysis. The second stage, ‘define and map policies’, corresponds to both requirements gathering & analysis and design phases in the SDLC. The third stage concerns how to process policies, which corresponds to the implementation phase in the SDLC, and the fourth phase, install and deploy, is related to the deployment and testing phase in the SDLC. Each phase of the methodology is iterate-able. Figure 2 presents the methodology consisting of four phases, which are detailed below:

Concept Development: In the first phase, the core functions required, along with the smart devices that can perform those core functions in the IoT collective, are listed. Furthermore, the environment where the smart devices need to be deployed, as well as the end-users with whom the targeted smart devices will interact, are also listed in this phase. For example, if the owner of a supermarket wants to stream live the environment and would like the items to be delivered to the vehicles parked outside, then these required core functions, along with the smart devices, i.e., smart cameras and robots, to be listed in this phase. The devices that can execute those core functions are smart cameras and robots, thus this phase will also include a list of the needed devices. Additionally, if robots must interact with customers and cameras must interact with staff, this step categorises all end-users with whom the devices will interact.

Define and Map Policies: The administrative policy rules must be defined in this phase after the core functions (or behaviours) of each smart device have been determined in the last phase. For example, what authorisation, obligation, and prohibition rules under an administrative policy are necessary if a robot is to execute its basic job of keeping track of products on a supermarket shelf? Socio-ethical policy rules are pre-defined as shown in Figure 2 because these rules are required to make a device behave in an ethical manner.

In the proposed methodology, a set of socio-ethical policy rules are pre-define, one or more of which might be applicable to a given application. Administrative rules, on the other hand, must be specified in order to regulate smart devices’ environment-specific behaviours. After the administrative policy rules have been determined, the end user’s demographics, such as age, ethnicity, gender, and so on, must be defined. To allow smart devices to function appropriately, the user’s demographics must be established. A senior who goes to the supermarket, for example, needs help locating milk. The elderly is taken to the milk by a robot that has been deployed at the store. Similarly, assuming two customers (one senior and the other adult) want the same help of locating the milk. A robot hands a map to a the adult customer and asks if he or she can go alone, then transports the senior person to the milk place. Smart devices can behave better based on the user’s demographics. Once the user’s demographics are defined, the socio-ethical and administrative policies need to be mapped to each defined core function of the device.

Both socio-ethical and administrative policies comprise authorisation, obligation, and prohibition rules, as stated in section one. As a result, each core function will be mapped to the policies’ particular authorisation, obligation, and prohibition rules. The idea is that the development team thinks about and writes out not only each device’s core functions/behaviours, but also the codes up applicable policy rules (as identified with stakeholders or clients) that relate to the device’s socio-ethical aspects (i.e., the device’s authorisations/prohibitions and obligations) while performing core functions.

Process Policies: Three policy documents created by the designer must be loaded onto smart devices in this phase, since they contain the policy rules to operationalise the authorisations/prohibitions and obligations mapped to the devices’ core functions/behaviours. The first document explains what policy rules a device has to follow, while the second and third documents explain how to interpret the socio-ethical and administrative policy rules, respectively. When each document is placed on smart devices, they will be able to learn what rules to follow and how to interpret each rule while carrying out their core job. Note that this work has defined what rules each device has to follow separately from how an applicable rule is interpreted by a device since two devices might interpret the same policy rule differently, depending on their core functions and capabilities.

Install: The final phase is to put each device into the environment after loading policy documents. If a robot is to be placed in a supermarket, for example, once the robot’s basic functions have been defined, policies have been mapped to each core function, and policy language documents have been embedded in the robot, it is finally ready to begin operation.

Each stage of the proposed methodology aids in the deployment of smart devices that have socially appropriate interaction behaviours. The behaviour of the devices prior to the application of policies is only a core function that they execute (which can be an absence of acceptable behaviour). The devices’ behaviour becomes ethical and controlled after mapping socio-ethical and administrative policies to the devices’ core functions and embedding them with policy rules to operationalise the right socio-ethical behaviours. In the next sections, it is discussed how the proposed methodology has been applied to the supermarket, children’s hospital, and early learning centre scenarios.

## 5. Application of the Methodology to Three Different Scenarios

Multiple smart devices, particularly robots, are being used in various areas without consideration for the regulation of their interaction behaviour [15,16,17]. Previously, a policy-based approach has been applied to robots to govern their interaction behaviours in a scenario of an elderly care facility [2,29], though without an elaboration of the methodology that has been proposed in this paper. As an illustration of the proposed methodology, it has been applied to a supermarket, children’s hospital and early learning centre scenarios here. The methodology is tested on three different scenarios because of the different targeted populations. In children’s hospitals as well as early learning centres, it has been observed that the kids as patients and the children as learners feel better and enjoy communicating with the robots [26]. human–device interaction is the focus of many studies, but child–robot/IoT interaction must also be considered. The proposed methodology in this paper is applicable to multiple domains, but three scenarios have been selected where different age groups of the population can be targeted.

### 5.1. Application of the Methodology to a Supermarket Scenario

**Figure 3:**
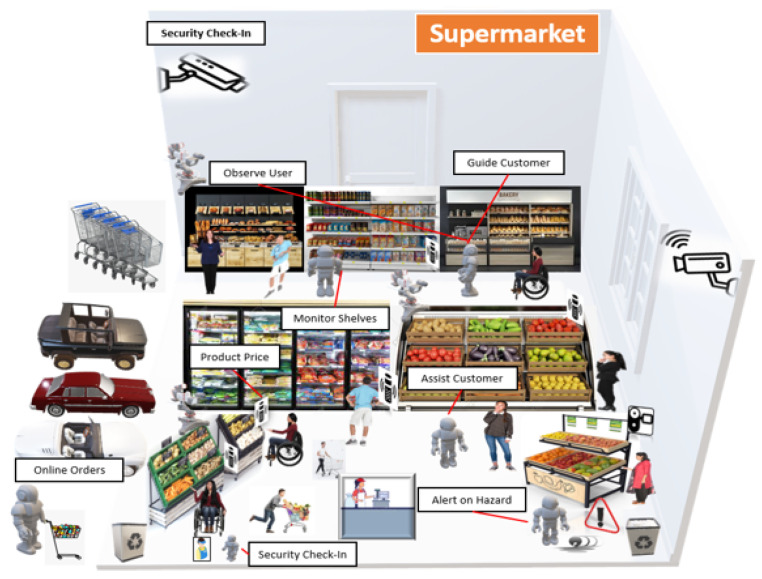
Supermarket domain showing an IoT collective consisting of multiple robots, smart cameras, smart speakers with customers and employees.

Figure 3 illustrates the whole supermarket scenario, in which a variety of smart devices, such as robots, smart cameras, and smart speakers, must be placed to execute their core functions. According to the scenario, various robots must be placed throughout the market to execute various tasks, including keeping track of the shelves, keeping an eye on customers, detecting rubbish on the floor, recognising staff members’ faces at the main door for attendance purposes, delivering online-ordered products to automobiles parked outside the market, assisting customers and directing them to various shop areas. Further, smart cameras must be put on the market: to keep an eye on the surroundings and stream live for security purposes. Finally, numerous smart speakers must be placed at various shop locations to allow customers to scan the product bar-code and obtain a description of the item. Following the methodological phases, the devices must perform activities adhering to policies that must be mapped to their core functions. Furthermore, devices will learn how to interpret each policy through policy documents.

#### Methodology’s Applicability

Each phase of the proposed methodology has been tested on the supermarket scenario explained above. The phases are described below:

Concept Development: Following the first phase of the methodology, Table 1 displays the core functions as well as the smart devices that perform those core functions in the supermarket environment. The targeted smart devices are actuators (IoT devices): robots, smart cameras, smart speakers, mediators (Communicating Devices): smartphones and tablets, and administrators (Admin Devices): smartphones and tablets.

**Figure 4:**
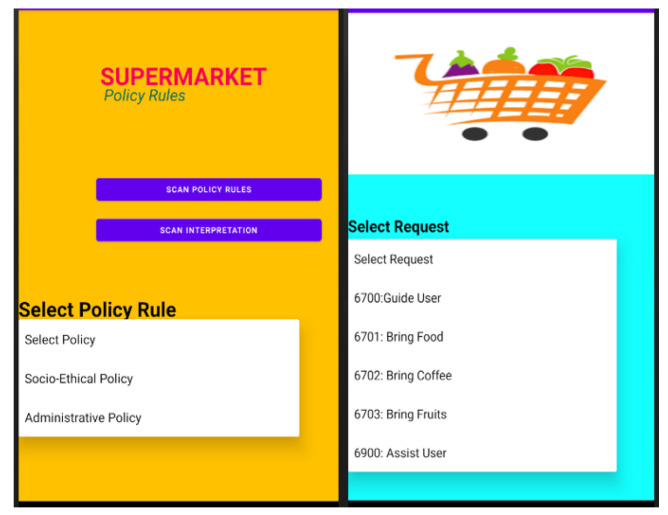
Administrator App (**Left**), Mediator App (**Right**).

The robots are using launcher OS 1337 and software version 120.07. Android OS is used in smart speakers and smart cameras. Similarly, Android OS is used on mediator and administrator devices. Figure 4 shows the mediator application’s interface, which users may utilise to interact with the robot, and the administrator’s application interface, which the admin may use to enforce administrative regulations on smart devices. The supermarket is the environment in which the smart devices must be installed, and the end-users with whom the devices will interact include customers, employees, and administrators.

Define and Map Policies: Following the second phase of the methodology, Table 2 and Table 3 outline the defined socio-ethical policies and administrative policies mapped to the devices’ core functions. The user demographic selected is the age group where the behaviour of devices, especially robots, can be improved. If a senior request that the robot takes him or her to the biscuit location while a young client requests that the robot show him or her the bread location, the robot must show the map to the young person while taking the senior to the biscuit site.

Process Policies: Following the third phase, the policy language documents are created independently for each targeted smart device. As a result, each device must load its three policy documents in order to understand what policy rules it must obey and how to interpret each socio-ethical and administrative policies while executing its core functions. For instance, if a robot has a core function of taking users to different locations, then the authorisation rule that applies to its core function is “guide-user". While guiding, the obligation rules that apply to robots are to “be-respectful", which means being polite with the users, “be-transparent-in-action", which means informing the users of the location where they are being directed by the robot, and “handle-uncertainty", which means avoiding obstacles or finding alternate paths in crowded areas. The prohibition applies is “moving out of range" (i.e., it should not move out of range) under the socio-ethical policy. The robot must know how to interpret these rules while guiding the user. Therefore, it is essential to load policy documents onto robots to let them understand the interpretation of each rule through the designed policy language documents. (The full document on robot socio-ethical policy is available: https://github.com/abatool-abatool/POLICY-LANGUAGE-SUPERMARKET-DOMAIN/blob/main/Socio-Ethical-Robot-Supermarket (accessed on 15 May 2022). Similarly, the complete document for the socio-ethical policy of smart cameras is available: https://github.com/abatool-abatool/POLICY-LANGUAGE-SUPERMARKET-DOMAIN/blob/main/Socio-Ethical-Camera-Supermarket (accessed on 15 May 2022), and the smart speaker socio-ethical policy document is available: https://github.com/abatool-abatool/POLICY-LANGUAGE-SUPERMARKET-DOMAIN/blob/main/Socio-Ethical-Speaker-Supermarket (accessed on 15 May 2022). The full document of the robot administrative policy is available: https://github.com/abatool-abatool/POLICY-LANGUAGE-SUPERMARKET-DOMAIN/blob/main/Administrative-Robot-Supermarket (accessed on 15 May 2022). Similarly, the complete document of the administrative policy for the smart camera is available: https://github.com/abatool-abatool/POLICY-LANGUAGE-SUPERMARKET-DOMAIN/blob/main/Administrative-Camera-Supermarket (accessed on 15 May 2022) and the smart speaker’s administrative policy document is available:https://github.com/abatool-abatool/POLICY-LANGUAGE-SUPERMARKET-DOMAIN/blob/main/Administrative-Speaker-Supermarket (accessed on 15 May 2022)).

**Figure 5:**
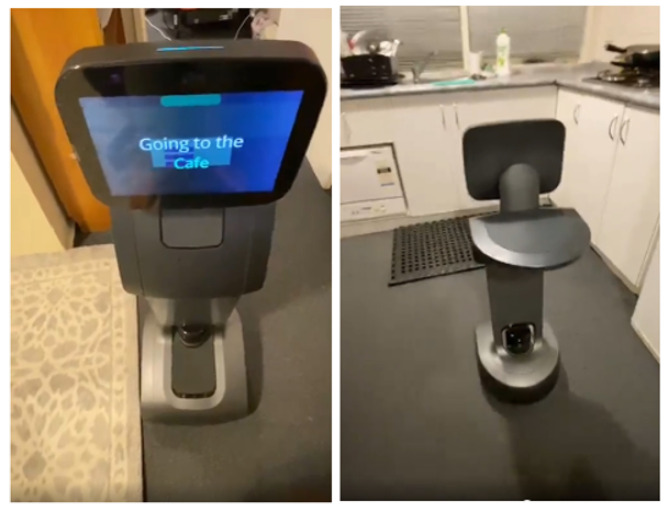
Robot guiding user.

Install: In this phase, each targeted device has to be installed in the supermarket. Figure 5 shows a robot taking a user’s guide-me request. When the robot receives the request, it goes to the spot where the user is and guides the user to where s/he wishes to go. Socio-ethical policies mapped to devices’ core functions work across domains, which implies that while a robot is guiding or aiding humans, it must follow socio-ethical policies, but administrative policies must be relevant to the environment. As a result, the administrator has the ability to enforce administrative policy rules on smart devices. The smart devices must obey all policy rules that apply to their core functionality when executing these activities. In terms of obligation rules, a smart camera, for example, is required to protect users’ privacy while viewing the surroundings, and it is not permitted to continue working if its power falls below the threshold in terms of prohibition rules under the socio-ethical policy. Similarly, the smart speaker is required to maintain loudness under administrative policy’s obligation rules and is not permitted to disturb customers needlessly under administrative policy’s prohibition rules.

A supermarket scenario is prototyped using Temi robots, smart cameras, and smart speakers as mentioned in the first phase of the methodology. Some of their core functions are successfully implemented, such as guiding users, delivering items, streaming live, and so on, and all policies have been applied to those specific core functions of the devices in this scenario.

### 5.2. Application of the Methodology to a Children’s Hospital Scenario

**Figure 6:**
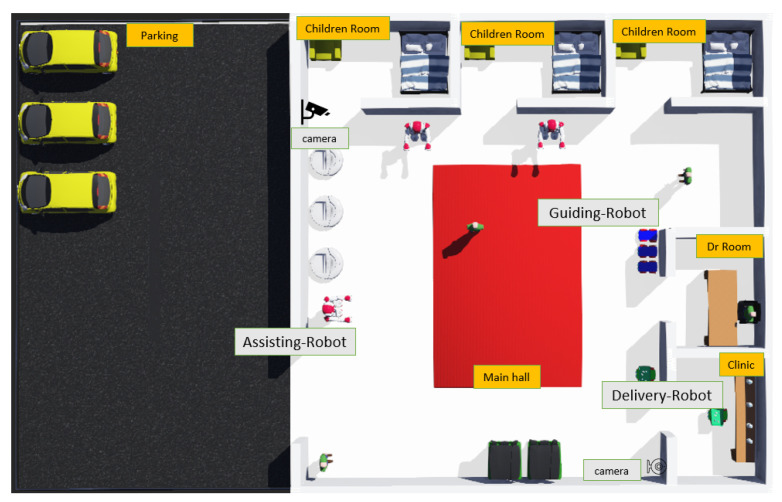
Children Hospital Domain showing an IoT collective consisting of multiple robots, smart cameras with patients and Dr.

Figure 6 presents the scenario of a children’s hospital, in which a variety of smart robots and smart cameras must be deployed to carry out their primary responsibilities. According to the scenario, three types of robots must be installed throughout the hospital to do various functions, including guiding, delivering, sanitising floors, displaying videos, monitoring, and assisting. The delivery robot transports medicines from the clinic to parked automobiles outside the hospital as well as patient rooms. The guidance robot directs individuals arriving at the hospital to various locations as well as sanitise the floors/walls, while the assisting robot is in charge of helping visitors in answering their queries as well as displaying media files (exercise videos and games) for children as required. Moreover, the assisting robot pass the patient health details to doctors on fixed time intervals. The smart cameras are placed for security purposes, displaying the captured videos to administration and streaming live to make sure everything is good around. Following the methodological phases, each device must carry out its core functions while adhering to policies that must be mapped to its basic functions. Furthermore, each device will learn to understand policy documents in order to interpret policies.

#### Methodology’s Applicability

**Figure 7:**
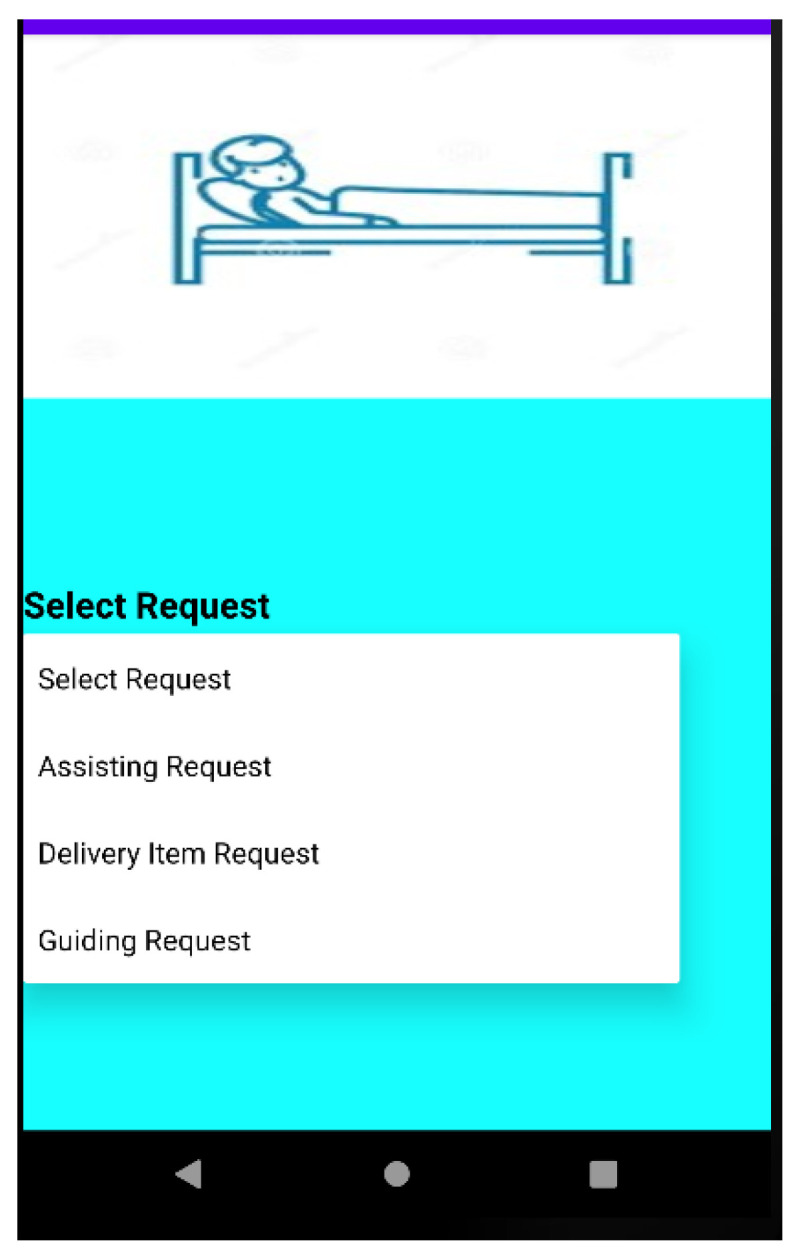
Mediator App.

The above-mentioned children’s hospital scenario has been used to test each phase of the proposed methodology. The phases are described below:

Concept Development: Following the first phase of the methodology, Table 4 displays the core functions performed by each device in terms of children’s hospital environment. The targeted smart devices are actuators (IoT devices): robots and smart cameras. Mediators (Communicating Devices): smartphones and tablets, and administrators (Admin Devices): smartphones and tablets. Figure 7 shows the mediator application’s interface, which users may use to interact with the robot. The children’s hospital is the environment in which the smart devices must be installed, and the end-users with whom the devices will interact include patients, doctors, staff members, and visitors.

Define and Map Policies: Table 5 and Table 6 outline the socio-ethical policies and administrative policies defined and mapped to the devices’ core functions. In the children’s hospital scenario, the user demographics chosen are age and birth country. The birth country is used so that smart devices, particularly robots, may speak with the child patient or staff in their native tongue. It is critical to communicate effectively in hospitals in order to better comprehend the problems of patients, and the age group is chosen according to the various age groups of children and employees (nurses, doctors). If a robot is required to play workout videos, it can, for example, display moderate aerobic motions for children under the age of six and various sorts of movements for children aged six and above, such as muscular strength, bone health, or dancing moves.

Process Policies: The policy documents must be processed by each device in the third step of the proposed methodology. As a result, in terms of the children’s hospital scenario, policy language documents are prepared separately for each targeted smart device in this step. Each device must load its three policy documents in order to understand what policy rules it must obey and how to interpret each socio-ethical and administrative policies while executing its core functions. For instance, if a robot has a core function of sanitising the floors of a hospital, then the authorisation rule that applies to its core function is “safety measures”. While sanitising, the obligation rules that apply to robots are to “be-transparent-in-action”, which means informing the users of the location which are fully sanitised, and “handle-uncertainty”, which means avoiding obstacles or finding alternate paths in crowded areas. The prohibition applies is “moving out of range” (i.e., it should not move out of range) under the socio-ethical policy. The robot must know how to interpret these rules while sanitising the floors of the hospital. Therefore, as before, it is essential to load policy documents onto robots to let them understand the interpretation of each rule through the designed policy language documents. (The full document on robot socio-ethical policy is available: https://github.com/abatool-abatool/POLICY_LANGUAGE_CHILDREN-S_HOSPITAL/blob/main/Socio-Ethical-Robot-Children’s-Hospital (accessed on 16 May 2022). Similarly, the complete document for the socio-ethical policy of smart cameras is available: https://github.com/abatool-abatool/POLICY_LANGUAGE_CHILDREN-S_HOSPITAL/blob/main/Socio-Ethical-Camera-Children’s-Hospital (accessed on 16 May 2022). The full document of the robot administrative policy is available: https://github.com/abatool-abatool/POLICY_LANGUAGE_CHILDREN-S_HOSPITAL/blob/main/Administrative-Robot-Children-Hospital (accessed on 16 May 2022). Similarly, the complete document of the administrative policy for the smart camera is available: https://github.com/abatool-abatool/POLICY_LANGUAGE_CHILDREN-S_HOSPITAL/blob/main/Administrative-Camera-Children-Hospital) (accessed on 16 May 2022).

**Figure 8:**
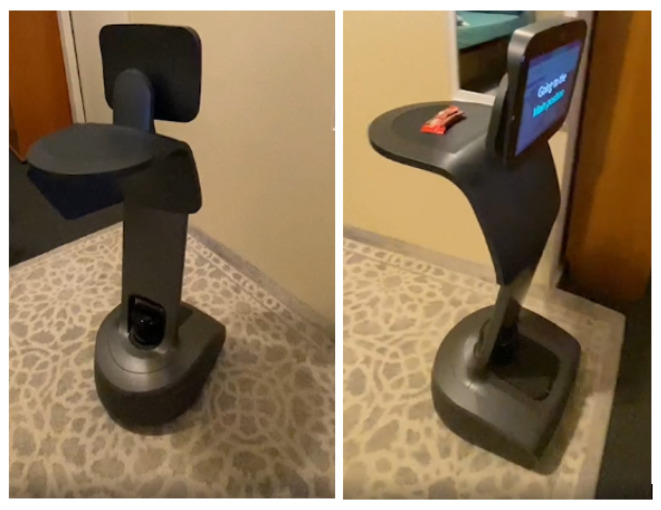
Robot completing delivering an item request.

Install: In this phase, each targeted device has to be installed in the hospital. Figure 8 shows a robot taking a user’s delivering an item request. When the robot receives the request, it goes to the spot to take the item and delivers it to the users’ location. Socio-ethical policies mapped to device fundamental functions work across domains, which implies that while a robot is delivering an item or assisting humans, it must follow socio-ethical policies, but administrative policies must be relevant to the environment. As a result, the administrator has the ability to enforce administrative policy rules on smart devices. The methodology was successfully applied to the children’s hospital situation, illustrating core functions and associated socio-ethical policies.

A children’s hospital scenario is prototyped using Temi robots, and smart cameras as mentioned in the first phase of the methodology. Some of their core functions are successfully implemented, such as guiding users, delivering items, displaying videos, streaming live, and so on, and all policies have been applied to those specific core functions of the devices in this scenario.

### 5.3. Application of the Methodology to an Early Learning Centre Scenario

**Figure 9:**
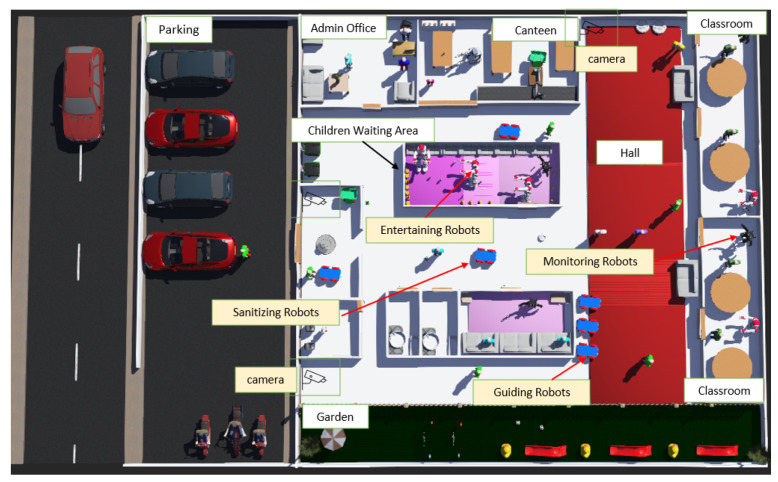
Early Learning Centre Domain showing an IoT collective consisting of multiple robots, smart cameras with teachers and visitors.

A care receiver (taken care of by children) robot for a classroom in an English-learning school is designed by Fumihide and Madhumita [30]. The care receiver robot has been built to decrease the ethical risks associated with smart technology. An experiment in one of Japan’s schools discovered that the care-receiver robot reduces some ethical issues and can be a high-impact reinforcement learning technique for children. Figure 9 illustrates the scenario of an early learning centre where the smart devices are deployed. As in the earlier scenarios, each device must carry out its core function and while doing so it must adhere to policies applied to them following the methodological phases.

According to the scenario, four types of smart robots and multiple smart cameras must be deployed to do various functions including entertaining kids in the playing area, monitoring their daily activities, playing educational games, and guiding to various locations of the centre. The robots are responsible of monitoring the children activities, entertaining them in playing area, playing educational games in learning classes, passing the children activity details to teachers, sanitising the walls and floors, interacting with visitors to see if they need any assistance and guiding the visitors to the offices as well as children to the class rooms. On the other hand, smart cameras are responsible for streaming live, displaying stream videos to administration to make sure everything is good around and alerting on hazards.

#### Methodology’s Applicability

Each phase of the proposed methodology is tested by applying to above mentioned early learning centre scenario. The application of each phase is described below:

Concept Development: Following the first phase of the methodology, Table 7 displays the core functions performed by each device in terms of early learning centre environment. The targeted smart devices are actuators (IoT devices): robots and smart cameras. Mediators (Communicating Devices): smartphones and tablets, and administrators (Admin Devices): smartphones and tablets. Figure 9 shows the mediator application’s interface, which users may use to interact with the robot, and the administrator’s application interface, which the admin may use to enforce administrative regulations on smart devices. The early learning centre is the environment in which the smart devices must be installed, and the end-users with whom the devices will interact include children, teachers, staff members, and visitors.

Define and Map Policies: Table 8 and Table 9 outlines the socio-ethical policies and administrative policies mapped to the devices’ core functions. Age, country of birth, and education are the user demographics chosen in the early learning setting. The country of birth is chosen so that smart devices, particularly robots, may converse with visitors in their native tongue. It is simple for a visitor to speak with the robot if he or she wants the robot to direct them to the administrative office. Because of the various age groupings of children, the age is selected. If a robot is required to play educational videos, it can display the appropriate educational video based on the age group (different classroom levels). Finally, education is chosen according to the teachers’ various degrees of education. If a robot has to convey the specifics of a child’s activities to the teacher, it does so depending on the teacher’s educational level and sends the information to the one who can review the child’s evaluations.

Process Policies: As each device has to process the policy documents in this phase. Therefore, each policy document prepared at this phase must be loaded by each device to understand the interpretation of each rule. The designed policy language documents can be found in this link. (The full document on robot socio-ethical policy is available: https://github.com/abatool-abatool/POLICY_LANGUAGE_EARLY-LEARNING-CENTRE/blob/main/Socio-Ethical-Robot-Early-Learning-Centre (accessed on 17 May 2022). Similarly, the complete document for the socio-ethical policy of smart cameras is available: https://github.com/abatool-abatool/POLICY_LANGUAGE_EARLY-LEARNING-CENTRE/blob/main/Socio-Ethical-Camera-Ealy-Learning-Centre (accessed on 16 May 2022). The full document of the robot administrative policy is available: https://github.com/abatool-abatool/POLICY_LANGUAGE_EARLY-LEARNING-CENTRE/blob/main/Administrative-Robot-Early-Learning-Centre (accessed on 17 May 2022). Similarly, the complete document of the administrative policy for the smart camera is available: https://github.com/abatool-abatool/POLICY_LANGUAGE_EARLY-LEARNING-CENTRE/blob/main/Administrative-Camera-Early-Learning-Centre (accessed on 17 May 2022)).

Install: In this phase, each device has to be installed in the early learning centre. Therefore, each device’s functionality is tested to evaluate the phases of the proposed methodology. The methodology was successfully applied to the early learning centre situation, illustrating core functions and associated socio-ethical policies.

Overall, it is highly beneficial to deploy IoT devices in various contexts while maintaining control over their interaction behaviour. The methodology was successfully applied to the supermarket, early learning centre and children’s hospital situation, illustrating core functions and associated socio-ethical policies. An early learning centre scenario has been prototyped using Temi robots only, but realised the functions and policies for the smart cameras. Some of the core functions of the robots are successfully implemented, such as guiding users, delivering items, and displaying videos, and all policies have been applied to those specific core functions of the devices in this scenario.

## 6. Discussion

The application of the methodology to different scenarios, i.e., supermarket, children’s hospital, and an early learning centre, assisted in developing the socio-ethical dimensions of the human-centred complex interaction system in which multiple devices perform their core functions while adhering to policies that require them to behave ethically and in more human-centric ways.

The methodology proposed in this research is designed in a way to assist developers from the design stage till the testing stage to come up with smart devices embedded with socio-ethical behaviours. To make it easier for developers to comprehend the ideas of each step, the phases of the suggested methodology are related to the stages of the software engineering methodology. Application of the proposed methodology to different smart devices (IoT collectives) in different environments with different age groups of the population has helped in identifying the applicability of the methodology. It is critically analysed that while mapping the socio-ethical policy rules with devices’ core functions, the proposed authorisation rules, which reflect the allowable tasks to be performed by the devices, are different for each core function, but the proposed obligation and prohibition rules (for the most part) are the same. The interesting part is that the pre-defined socio-ethical policy rules have been reused from supermarket scenario to the children’s hospital as well as the early learning centre scenarios. Furthermore, the devices in each scenario with the same policy rules have shown similar interpretations, but the devices themselves have shown different interpretations from one another. The safety measures policy mapped to the robot’s core functions has, for example, been interpreted differently by the smart camera—i.e., same (high-level) policy on two devices but different interpretations (realisations), providing a level of abstraction.

Instead of developing apps that allow the devices to fulfil their required functions solely, the suggested methodology allows the project team to develop socio-ethical smart device applications. Following the stages from requirement gathering to testing allows the project team to develop software applications with specific user requirement features in the traditional software engineering approach. However, the proposed methodology in this paper allows the team to come up with not only the device core functions to perform but also socio-ethical embedded policies that allow devices to behave in more human-centric ways. As the pre-defined socio-ethical policies are reused in the scenarios, it also helps the project team not to come up with policies again and again. However, the suggested user demographics phase in the methodology’s “define and map policies” stage should be assessed to determine if it has a good influence on device behaviour.

The application of the proposed methodology to three different scenarios demonstrates the versatility of the approach. Note that the policies that this paper have illustrated are mainly examples and in different environments, preferences, and circumstances, there would be different applicable policies required.

## 7. Conclusions and Future Work

As devices acquire more agency, become smarter, and may be utilised by many users, the technique of applying policies to IoT collectives to govern their interaction behaviours during human–device interaction is necessary. A four-phase methodology for developing and deploying IoT devices that exhibit socially acceptable behaviours is proposed in this paper. The methodology’s application is demonstrated in a supermarket, children’s hospital and an early learning centre setting, where each device adheres to socio-ethical and administrative policies in order to interact with users appropriately and ethically. It has been shown that certain socio-ethical behaviours can be abstracted as policy rules and considered separately from device core functions, rather than being mixed in with core functions—a useful separation of concerns from a software engineering standpoint, especially when designing for collections of smart IoT devices with increasing agency. Future work will consider the methodology for more IoT environments and the prototype will further develop.

For illustration purposes, the proposed methodology is applied to the smart devices in three different settings. From the illustration provided in the paper, it has been observed that machines act in more human-centric ways while carrying out their essential tasks and that they adhere to socio-ethical norms. Devices may encounter difficulties when carrying out their responsibilities, such as moving out of range, colliding with one another, capturing a person’s information without that person’s authorisation, hitting people by moving closer to them, and so forth. The application of the methodology to the smart devices caused them to act morally and with a greater focus on people. By doing this, the difficulties are lessened and the machines exhibit moral behaviour. Moreover, our approach provides separation of concerns between core functions and socio-ethical requirements.

## Figures and Tables

**Table 1:** Core Functions of Smart Devices in Supermarket Scenario.

Smart Device	Core Functions
Cleaning Robot	Detect litter/trash on the floor to prevent customers from slipping and getting hurt
Monitoring Robot	Recognise staff members’ faces to record their attendance
	Keeps track of products on shelves
	Keeps an eye on the customers to make sure everything is in order
Delivery Robot	Deliver online-ordered products to cars parked outside the store
Assisting Robot	Interact with customers as they enter the store to see whether they require assistance
	Display item details to customers to let them know the item price, expiry date, and so on
Guiding Robot	Take customers to various locations to direct them to the products on the shelves
Smart Security Camera	For security reasons, record footage of the surroundings
	Recognise the faces of employees before displaying video to them
Smart Monitoring Camera	Detect dangers by streaming the environment in real-time
	Alert about hazards/issues with network staff
	Look after the customers to make sure everything is good around
Smart Assisting Speaker	Display product prices to customers
	Scan product bar-codes to keep track of items
Smart Security Speaker	Detect staff members’ voices for security purposes
	Alert about hazards/issues with items to staff
Smart Entertaining Speaker	Play music in cafe areas for visitors
	Tell Jokes in cafe areas for entertainment purposes

**Table 2:** Socio-ethical policies and core functions mapping—supermarket scenario.

Smart Device	Core Function	Authorisation	Obligation	Prohibition
Assisting Robot	Interacting with users	Communicate-with-Users	Be-Respectful	Harm-User
			Be-Transparent-in-Actions	Stay-With-Low-power
Monitoring Robot	Recognise users’ face	Security-Check-In	Be-Privacy-Respecting	Harm-User
			Be-Prudent	Stay-With-Low-power
			Secure-Data	
			Be-Transparent-in-Actions	
Guiding Robot	Take users to different locations	Guide-Users	Handle-Uncertainty	Move-Out-of-Range
			Be-Respectful	Stay-With-Low-Power
			Be-Transparent-in-Actions	Have-Long-Communication
Cleaning Robot	Detects trash on floor	Safety-Measures	Be-Respectful	Harm-User
			Handle-Uncertainty	Move-Out-of-Range
				Stay-With-Low-power
Smart Security Camera	Capture videos/photos	Safety-Measures	Be-Privacy-Respecting	Harm-Users
			Be-Transparent-in-Actions	Move-Out-of-Range
			Handle-Task-Failure	Stay-With-Low-Power
				Have-Long-Communication
Smart Security Camera	Display video/photo	Communicate-with-Users	Be-Respectful	Stay-With-Low-Power
			Transparent-in-Actions	Have-Long-Communication
			Secure-Data	Harm-Users
			Handle-Task-Failure	
Smart Security Camera	Stream live	Safety-Measures	Be-Privacy-Respecting	Harm-Users
			Be-Transparent-in-Actions	Move-Out-of-Range
			Be-Prudent	Stay-With-Low-Power
			Handle-Task-Failure	Have-Long-Communication
Smart Security Camera	Detect user’s face	Security-Check-In	Be-Respectful	Stay-With-Low-power
			Be-Prudent	Harm-Users
			Secure-Data	
			Be-Transparent-in-Actions	
Smart Entertaining Speaker	Playing music	Guide-Users	Be-Privacy-Respecting	Harm-Users
		Safety-Measures	Be-Transparent-in-Actions	Move-Out-of-Range
			Handle-Task-Failure	Stay-With-Low-Power
			Be-Prudent	Have-Long-Communication
Smart Entertaining Speaker	Telling jokes	Communicate-with-Users	Be-Respectful	Harm-Users
		Safety-Measures	Be-Transparent-in-Actions	Stay-With-Low-Power
			Handle-Task-Failure	Have-Long-Communication
Smart Security Speaker	Detect users’ voice	Security-Check-In	Be-Respectful	Stay-With-Low-power
			Be-Prudent	Harm-Users
			Secure-Data	
			Be-Transparent-in-Actions	
Smart Assisting Speaker	Display product prices	Communicate-with-Users	Be-Respectful	Harm-Users
			Be-Transparent-in-Actions	Stay-With-Low-Power
				Have-Long-Communication

**Table 3:** Administrative policy rules and core functions mapping—supermarket scenario.

Smart Device	Core Function	Authorisation	Obligation	Prohibition
Monitoring Robot	Overlook users	Observe-User	Protect-Self	Enter-Private-Locations
			Be-Prompt-In-Actions	Talk-To-Strangers
			Alert-To-Fire-Alarms	
Assisting Robot	Display item details to user	Assist-User	Maintain-Volume	Enter-Private-Locations
		Enlist-Product-Prices	Be-Prompt-In-Actions	Talk-To-Strangers
			Protect-Self	Disturb-Unnecessarily
Monitoring Robot	Keep track of products in shelves	Monitor-Shelves	Maintain-Volume	Enter-Private-Locations
			Be-Prompt-In-Actions	Talk-To-Strangers
Delivery Robot	Manage Electronic Shopping	Manage-Online-Orders	Maintain-Volume	Talk-To-Strangers
			Be-Attentive	Disturb- Unnecessarily
				Enter-Private-Locations
Smart Monitoring Camera	Overlook users	Observe-User	Maintain-Volume	Talk-To-Strangers
			Be-Prompt-In-Actions	
Smart Security Camera	Detect user’s face	Observe-User	Maintain-Volume	Talk-To-Strangers
			Be-Prompt-In-Actions	Disturb- Unnecessarily
			Be-Attentive	
Smart Monitoring Camera	Alert on hazard	Assist-User	Alert-To-Fire-Alarms	NA
			Be-Prompt-In-Actions	
Smart Assisting Speaker	Scan barcodes	Assist-User	Maintain-Volume	Talk-To-Strangers
		Enlist-Product-Prices		Disturb- Unnecessarily
Smart Security Speaker	Detect users’ voice	Observe-User	Maintain-Volume	Talk-To-Strangers
			Be-Prompt-In-Actions	Disturb- Unnecessarily
			Be-Attentive	
Smart Security Speaker	Alert on hazard	Assist-User	Alert-To-Fire-Alarms	NA
			Be-Prompt-In-Actions	

**Table 4:** Core functions of smart devices in children’s hospital scenario.

Smart Device	Core Functions
Sanitising Robot	Sanitise the floors for safety purposes
Monitoring Robot	Monitor patients and send the patients’ health data to doctors
Delivery Robot	Deliver medicines to the patient rooms and cars parked outside the hospital
Assisting Robot	Interact with visitors as they enter the hospital to see whether they require assistance
	Display exercise and game videos to patients (children)
Guiding Robot	Take patients and visitors to various locations of the hospital
Smart Security Camera	For security reasons, record footage of the surroundings
	Recognise the faces of employees before displaying video to them
Smart Monitoring Camera	Detect dangers by streaming the environment in real-time
	Alert about hazards/issues with network staff
	Look after the patients to make sure everything is good around

**Table 5:** Socio-ethical policies and core functions mapping—children’s hospital.

Smart Device	Core Function	Authorisation	Obligation	Prohibition
Assisting Robot	Interacting with users	Communicate-with-Users	Be-Respectful	Harm-User
			Be-Transparent-in-Actions	Stay-With-Low-power
Sanitising Robot	Sanitise walls and floors	Safety-Measures	Handle-Uncertainty	Harm-User
			Be-Prudent	Stay-With-Low-power
			Be-Transparent-in-Actions	Move-Out-of-Range
Monitoring Robot	Transfer User’s Data	Safety-Measures	Handle-Uncertainty	Stay-With-Low-power
			Secure-Data	
Guiding Robot	Take users to different locations	Guide-Users	Handle-Uncertainty	Move-Out-of-Range
			Be-Respectful	Stay-With-Low-Power
			Be-Transparent-in-Actions	Have-Long-Communication
Assisting Robot	Display Media	Communicate-with-Users	Handle-Uncertainty	Move-Out-of-Range
			Be-Respectful	Stay-With-Low-Power
			Be-Transparent-in-Actions	Have-Long-Communication
Delivery Robot	Deliver Items to the locations	Safety-Measures	Be-Respectful	Harm-User
			Handle-Uncertainty	Move-Out-of-Range
				Stay-With-Low-power
Smart Security Camera	Capture videos/photos	Safety-Measures	Be-Privacy-Respecting	Harm-Users
			Be-Transparent-in-Actions	Move-Out-of-Range
			Handle-Task-Failure	Stay-With-Low-Power
				Have-Long-Communication
Smart Security Camera	Display video/photo	Communicate-with-Users	Be-Respectful	Stay-With-Low-Power
			Transparent-in-Actions	Have-Long-Communication
			Secure-Data	Harm-Users
			Handle-Task-Failure	
Smart Security Camera	Stream live	Safety-Measures	Be-Privacy-Respecting	Harm-Users
			Be-Transparent-in-Actions	Move-Out-of-Range
			Be-Prudent	Stay-With-Low-Power
			Handle-Task-Failure	Have-Long-Communication

**Table 6:** Administrative policy rules and core functions mapping—children’s hospital.

**Smart Device**	**Core Function**	**Authorisation**	**Obligation**	**Prohibition**
Monitoring Robot	Overlook users	Observe-User	Protect-Self	Enter-Private-Locations
			Be-Prompt-In-Actions	Talk-To-Strangers
			Alert-To-Fire-Alarms	
Assisting Robot	Display exercise videos to user	Assist-User	Maintain-Volume	Enter-Private-Locations
			Protect-Self	Disturb-Unnecessarily
			Be-Prompt-In-Actions	Talk-To-Strangers
Assisting Robot	Play games for children	Assist-User	Maintain-Volume	Enter-Private-Locations
			Protect-Self	Disturb-Unnecessarily
			Be-Prompt-In-Actions	Talk-To-Strangers
Monitoring Robot	Transfer User’s Health Data	Assist-User	Protect-Self	Talk-To-Strangers
Smart Monitoring Camera	Overlook users	Observe-User	Maintain-Volume	Talk-To-Strangers
			Be-Prompt-In-Actions	
Smart Security Camera	Display Video to Admin	Assist-User	Maintain-Volume	Talk-To-Strangers
			Disturb- Unnecessarily	
Smart Monitoring Camera	Alert on hazard	Assist-User	Alert-To-Fire-Alarms	NA
			Be-Prompt-In-Actions	

**Table 7:** Core functions of smart devices in early learning scenario.

Smart Device	Core Functions
Sanitising Robot	Sanitise the floors for safety purposes
Monitoring Robot	Monitor children activities and pass the details to the teachers
Entertaining Robot	Play with children in playing area
Assisting Robot	Interact with visitors to see whether they require assistance
	Display educational game for children in the classrooms
Guiding Robot	Take children and visitors to various locations of the centre
Smart Security Camera	For security reasons, record footage of the surroundings
	Recognise the faces of employees before displaying video to them
Smart Monitoring Camera	Detect dangers by streaming the environment in real-time
	Alert about hazards/issues with network staff
	Look after the children to make sure everything is good around

**Table 8:** Socio-ethical policies and core functions mapping—early learning centre.

Smart Device	Core Function	Authorisation	Obligation	Prohibition
Assisting Robot	Interacting with users	Communicate-with-Users	Be-Respectful	Harm-User
			Be-Transparent-in-Actions	Stay-With-Low-power
Sanitising Robot	Sanitise walls and floors	Safety-Measures	Handle-Uncertainty	Harm-User
			Be-Prudent	Stay-With-Low-power
			Be-Transparent-in-Actions	Move-Out-of-Range
Monitoring Robot	Transfer User’s Data	Safety-Measures	Handle-Uncertainty	Stay-With-Low-power
			Secure-Data	
Guiding Robot	Take users to different locations	Guide-Users	Handle-Uncertainty	Move-Out-of-Range
			Be-Respectful	Stay-With-Low-Power
			Be-Transparent-in-Actions	Have-Long-Communication
Assisting Robot	Display Media	Communicate-with-Users	Handle-Uncertainty	Move-Out-of-Range
			Be-Respectful	Stay-With-Low-Power
			Be-Transparent-in-Actions	Have-Long-Communication
Entertaining Robot	Playing with user	Safety-Measures	Be-Respectful	Harm-User
			Handle-Uncertainty	Move-Out-of-Range
				Stay-With-Low-power
Smart Security Camera	Capture videos/photos	Safety-Measures	Be-Privacy-Respecting	Harm-Users
			Be-Transparent-in-Actions	Move-Out-of-Range
			Handle-Task-Failure	Stay-With-Low-Power
				Have-Long-Communication
Smart Security Camera	Display video/photo	Communicate-with-Users	Be-Respectful	Stay-With-Low-Power
			Transparent-in-Actions	Have-Long-Communication
			Secure-Data	Harm-Users
			Handle-Task-Failure	
Smart Security Camera	Stream live	Safety-Measures	Be-Privacy-Respecting	Harm-Users
			Be-Transparent-in-Actions	Move-Out-of-Range
			Be-Prudent	Stay-With-Low-Power
			Handle-Task-Failure	Have-Long-Communication

**Table 9:** Administrative policy rules and core functions mapping—early learning centre.

Smart Device	Core Function	Authorisation	Obligation	Prohibition
Monitoring Robot	Overlook users	Observe-User	Protect-Self	Enter-Private-Locations
			Be-Prompt-In-Actions	Talk-To-Strangers
			Alert-To-Fire-Alarms	
Assisting Robot	Display educational games to user	Assist-User	Maintain-Volume	Enter-Private-Locations
			Protect-Self	Disturb-Unnecessarily
			Be-Prompt-In-Actions	Talk-To-Strangers
Entertaining Robot	Play with children	Assist-User	Maintain-Volume	Enter-Private-Locations
			Protect-Self	Disturb-Unnecessarily
			Be-Prompt-In-Actions	Talk-To-Strangers
Monitoring Robot	Transfer Children’s Data	Assist-User	Protect-Self	Talk-To-Strangers
Smart Security Camera	Overlook users	Observe-User	Maintain-Volume	Talk-To-Strangers
			Be-Prompt-In-Actions	
	Display Video to Admin	Assist-User	Maintain-Volume	Talk-To-Strangers
			Disturb- Unnecessarily	
	Alert on hazard	Assist-User	Alert-To-Fire-Alarms	NA
			Be-Prompt-In-Actions	

## Data Availability

Supermarket: The full document on robot socio-ethical policy is available: https://github.com/abatool-abatool/POLICY-LANGUAGE-SUPERMARKET-DOMAIN/blob/main/Socio-Ethical-Robot-Supermarket (accessed on 16 May 2022). Similarly, the complete document for the socio-ethical policy of smart cameras is available: https://github.com/abatool-abatool/POLICY-LANGUAGE-SUPERMARKET-DOMAIN/blob/main/Socio-Ethical-Camera-Supermarket (accessed on 16 May 2022), and the smart speaker socio-ethical policy document is available: https://github.com/abatool-abatool/POLICY-LANGUAGE-SUPERMARKET-DOMAIN/blob/main/Socio-Ethical-Speaker-Supermarket (accessed on 16 May 2022). The full document of the robot administrative policy is available: https://github.com/abatool-abatool/POLICY-LANGUAGE-SUPERMARKET-DOMAIN/blob/main/Administrative-Robot-Supermarket (accessed on 17 May 2022). Similarly, the complete document of the administrative policy for the smart camera is available: https://github.com/abatool-abatool/POLICY-LANGUAGE-SUPERMARKET-DOMAIN/blob/main/Administrative-Camera-Supermarket (accessed on 17 May 2022) and the smart speaker’s administrative policy document is available: https://github.com/abatool-abatool/POLICY-LANGUAGE-SUPERMARKET-DOMAIN/blob/main/Administrative-Speaker-Supermarket (accessed on 17 May 2022). Children Hospital: The full document on robot socio-ethical policy is available: https://github.com/abatool-abatool/POLICY_LANGUAGE_CHILDREN-S_HOSPITAL/blob/main/Socio-Ethical-Robot-Children’s-Hospital (accessed on 16 May 2022). Similarly, the complete document for the socio-ethical policy of smart cameras is available: https://github.com/abatool-abatool/POLICY_LANGUAGE_CHILDREN-S_HOSPITAL/blob/main/Socio-Ethical-Camera-Children’s-Hospital (accessed on 16 May 2022). The full document of the robot administrative policy is available: https://github.com/abatool-abatool/POLICY_LANGUAGE_CHILDREN-S_HOSPITAL/blob/main/Administrative-Robot-Children-Hospital (accessed on 16 May 2022). Similarly, the complete document of the administrative policy for the smart camera is available: https://github.com/abatool-abatool/POLICY_LANGUAGE_CHILDREN-S_HOSPITAL/blob/main/Administrative-Camera-Children-Hospital (accessed on 17 May 2022). Early Learning Centre: The full document on robot socio-ethical policy is available: https://github.com/abatool-abatool/POLICY_LANGUAGE_EARLY-LEARNING-CENTRE/blob/main/Socio-Ethical-Robot-Early-Learning-Centre (accessed on 17 May 2022). Similarly, the complete document for the socio-ethical policy of smart cameras is available: https://github.com/abatool-abatool/POLICY_LANGUAGE_EARLY-LEARNING-CENTRE/blob/main/Socio-Ethical-Camera-Ealy-Learning-Centre (accessed on 16 May 2022).The full document of the robot administrative policy is available: https://github.com/abatool-abatool/POLICY_LANGUAGE_EARLY-LEARNING-CENTRE/blob/main/Administrative-Robot-Early-Learning-Centre (accessed on 17 May 2022). Similarly, the complete document of the administrative policy for the smart camera is available: https://github.com/abatool-abatool/POLICY_LANGUAGE_EARLY-LEARNING-CENTRE/blob/main/Administrative-Camera-Early-Learning-Centre (accessed on 17 May 2022).
